# 1′-Methyl-4′-phenyl-2′′-sulfanylidene­dispiro­[indoline-3,2′-pyrrolidine-3′,5′′-1,3-thia­zolidine]-2,4′′-dione

**DOI:** 10.1107/S1600536811056236

**Published:** 2012-01-11

**Authors:** Sampath Natarajan, Rita Mathews

**Affiliations:** a Department of Advanced Technology Fusion, Konkuk University, 1 Hwayang-dong, Gwangjin-gu, Seoul 143 701, Republic of Korea

## Abstract

The title compound, C_20_H_17_N_3_O_2_S_2_, crystallizes with two mol­ecules in the asymmetric unit. The pyrrolo­dine rings have envelope conformations in both mol­ecules, the N atoms deviating by 0.574 (3) and 0.612 (2) Å from the mean planes through the other ring atoms. The 1′-methyl and 4′-phenyl groups on the pyrrolidine rings are substituted in equatorial positions. In the crystal, mol­ecules are linked into a three-dimensional network by N—H⋯O, N—H⋯N and C—H⋯O and N—H⋯π hydrogen bonds.

## Related literature

The spiro­pyrrolidinyloxindole ring system is a frequently observed structural motif in many of the pharmacologically relevant alkaloids, see: Hilton *et al.* (2000 ▶). For the biological activity of heterocyclic compounds, see: Chavan *et al.* (2001 ▶); Baldwin *et al.* (1994 ▶); Amal Raj *et al.* (2003 ▶); Okita & Isobe (1994 ▶); Mogilaiah *et al.* (2001 ▶). For puckering parameters, see: Cremer & Pople (1975 ▶) and for asymmetry parameters, see: Nardelli (1995 ▶). For the synthesis, see: Sampath *et al.* (2010 ▶).
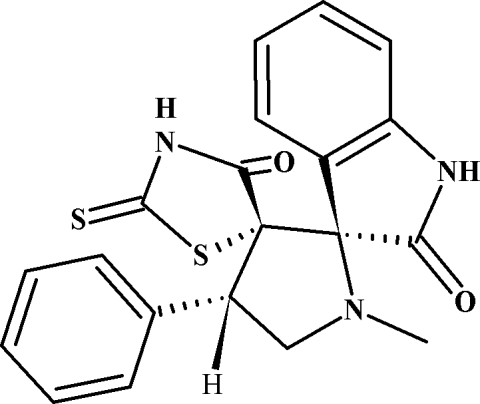


## Experimental

### 

#### Crystal data

C_20_H_17_N_3_O_2_S_2_
*M* *_r_* = 395.49
Monoclinic, 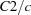

*a* = 24.259 (6) Å
*b* = 13.359 (3) Å
*c* = 23.628 (5) Å
β = 90.418 (7)°
*V* = 7657 (3) Å^3^
*Z* = 16
Mo *K*α radiation
μ = 0.30 mm^−1^
*T* = 293 K
0.45 × 0.38 × 0.24 mm

#### Data collection

Bruker SMART APEX CCD area-detector diffractometer
Absorption correction: refined from Δ*F* (*XABS*; Parkin *et al.* 1995 ▶) *T*_min_ = 0.703, *T*_max_ = 0.999
9064 measured reflections
9064 independent reflections
6169 reflections with *I* > 2σ(*I*)

#### Refinement

*R*[*F* ^2^ > 2σ(*F* ^2^)] = 0.071
*wR*(*F* ^2^) = 0.155
*S* = 1.09
9064 reflections
463 parameters
H-atom parameters constrained
Δρ_max_ = 0.54 e Å^−3^
Δρ_min_ = −0.49 e Å^−3^

### 

Data collection: *SMART* (Bruker, 2004 ▶); cell refinement: *SAINT* (Bruker, 2004 ▶); data reduction: *SAINT*; program(s) used to solve structure: *SHELXS97* (Sheldrick, 2008 ▶); program(s) used to refine structure: *SHELXL97* (Sheldrick, 2008 ▶); molecular graphics: *ORTEP-3* (Farrugia, 1997 ▶); software used to prepare material for publication: *PLATON* (Spek, 2009 ▶).

## Supplementary Material

Crystal structure: contains datablock(s) I, global. DOI: 10.1107/S1600536811056236/go2041sup1.cif

Structure factors: contains datablock(s) I. DOI: 10.1107/S1600536811056236/go2041Isup2.hkl

Additional supplementary materials:  crystallographic information; 3D view; checkCIF report

## Figures and Tables

**Table 1:** Hydrogen-bond geometry (Å, °) *Cg*1 is the centroid of the C9*B*–C14*B* ring.

*D*—H⋯*A*	*D*—H	H⋯*A*	*D*⋯*A*	*D*—H⋯*A*
N2*B*—H2*B*⋯O2*A*	0.86	2.17	2.794 (3)	129
N6*A*—H6*A*⋯N1*B*^i^	0.86	2.28	3.083 (3)	156
C4*B*—H4*B*⋯O1*A*^ii^	0.98	2.36	3.290 (3)	159
N6*B*—H6*B*⋯O1*A*^iii^	0.86	2.18	2.836 (3)	133
N2*A*—H2*A*⋯*Cg*1^iv^	0.86	2.95	3.806 (3)	170

Symmetry codes: (i) 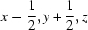; (ii) 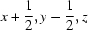; (iii) 
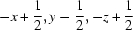
; (iv) 
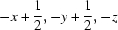
.

**Fig. 1.**
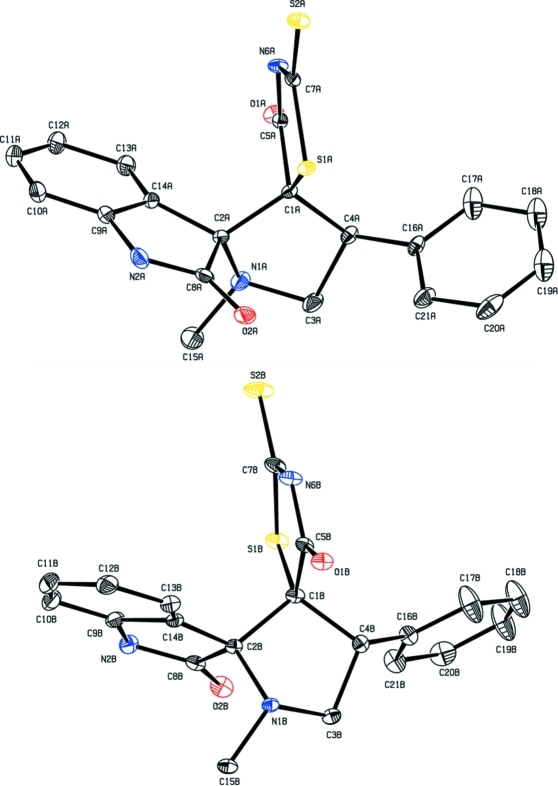
ORTEP diagram of the title molecule with the atom numbering scheme. Displacement ellipsoid are drawn at 30% probability level. H-atoms were removed for clarity.

**Fig. 2.**
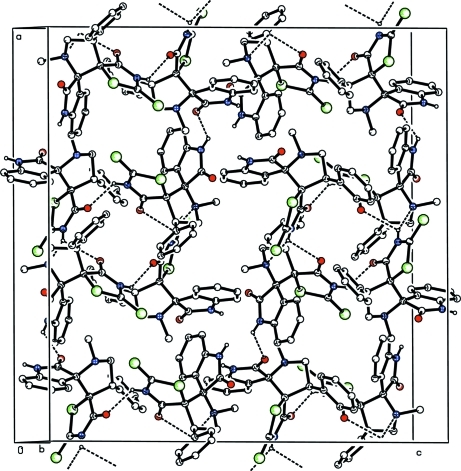
Packing diagram of the title compound viewed down the b axis. Dashed lines indicate the intra and intermolecular interactions between the molecules.

## supplementary crystallographic information

### Comment

Heterocyclic compounds are of interest because of their diverse biological activities (Chavan *et al.*, 2001; Baldwin *et al.*, 1994). Some received considerable attention because of their potential antimicrobial activity (Amal Raj *et al.*, 2003), especially indoles and spiroindoles, which play important roles in medicinal chemistry (Mogilaiah *et al.*, 2001). The spiropyrrolidinyloxindole ring system is a frequently observed structural motif in many of the pharmacologically relevant alkaloids (Hilton *et al.*, 2000). The derivatives of spirooxindole possess wide range of biological properties such as antimicrobial, antitumoral, antibiotic agents and inhibitors of human NK-1 receptor (Okita & Isobe, 1994).

The title compound (Fig. 1) crystallizes with two molecules (A and B) in the assymetric unit. Both these molecules contain a central of pyrrolidine ring, which is connected by two spiro junctions at the atoms C1 and C8 to a thiosolidine and oxindole rings, respectively. The pyrrolidine rings adopt an envelope conformation in both the molecules and the atoms N1A and N1B deviate -0.574 (3) and -0.612 (2) Å, respectively from the mean plane defined by the atoms C1, C2, C3 and C4. The other two substituents, the phenyl ring and methyl groups are oriented equatorially to best plane of the pyrrolidine rings.

The dihedral angles between the pyrrolidine ring and the oxidole and the thiosolidines moieties are 84.7 (1) and 88.2 (1)°, respectively for molecule A and 81.8 (1)and 90.0 (1)°, respectively for molecule B. The packing diagram of the title molecule viewed down *b* axis is shown in Fig. 2. The molecules are linked to form a three-dimensional network by N–H···O, N—H···N and C—H···O intra and intermolecular hydrogen bonds. In addition there is an N—H···π weak interaction, Table 1.

### Experimental

A mixture of 5-benzylidene-2-thioxo-1,3-thiazolidin-4-one (1 mmol), isatin (1 mmol) and sarcosine (1 mmol) were taken up in 20 ml of aqueous methanol and refluxed for 8 h on a water bath (Sampath *et al.*, 2010). The resultant product was subjected to column chromatography to yield the title compound and it was crystallized using methanol by slow evaporation method.

### Refinement

H atoms were positioned geometrically and refined using a riding model with C—H = 0.93 Å for aromatic H, 0.97 Å for methylene, 0.96 Å for methyl H atoms and for aromatic N—H = 0.86 Å. The *U*_iso_ parameters for H atoms were constraned to be 1.5Ueq of the carrier atom for the methyl H atoms and 1.2Ueq of the carrier atom for the remaining H atoms. The thermal and bond length parameters of the phenyl rings indicate that these rings are quite mobile.

### Crystal data

**Table:**

C_20_H_17_N_3_O_2_S_2_	*F*(000) = 3296
*M**_r_* = 395.49	*D*_x_ = 1.372 Mg m^−^^3^
Monoclinic, *C*2/*c*	Mo *K*α radiation, λ = 0.71073 Å
*a* = 24.259 (6) Å	Cell parameters from 9064 reflections
*b* = 13.359 (3) Å	θ = 1–28°
*c* = 23.628 (5) Å	µ = 0.30 mm^−^^1^
β = 90.418 (7)°	*T* = 293 K
*V* = 7657 (3) Å^3^	Lath, yellow
*Z* = 16	0.45 × 0.38 × 0.24 mm

### Data collection

**Table:**

Bruker SMART APEX CCD area-detector diffractometer	9064 independent reflections
Radiation source: fine-focus sealed tube	6169 reflections with *I* > 2σ(*I*)
graphite	*R*_int_ = 0.0000
ω scans	θ_max_ = 28.0°, θ_min_ = 1.7°
Absorption correction: part of the refinement model (Δ*F*) (*XABS*; Parkin *et al.* 1995).	*h* = −31→32
*T*_min_ = 0.703, *T*_max_ = 0.999	*k* = 0→17
9064 measured reflections	*l* = 0→31

### Refinement

**Table:**

Refinement on *F*^2^	Primary atom site location: structure-invariant direct methods
Least-squares matrix: full	Secondary atom site location: difference Fourier map
*R*[*F*^2^ > 2σ(*F*^2^)] = 0.071	Hydrogen site location: inferred from neighbouring sites
*wR*(*F*^2^) = 0.155	H-atom parameters constrained
*S* = 1.09	*w* = 1/[σ^2^(*F*_o_^2^) + (0.0495*P*)^2^ + 12.055*P*] where *P* = (*F*_o_^2^ + 2*F*_c_^2^)/3
9064 reflections	(Δ/σ)_max_ < 0.001
463 parameters	Δρ_max_ = 0.54 e Å^−^^3^
0 restraints	Δρ_min_ = −0.49 e Å^−^^3^

### Special details

**Table:**

Geometry. All e.s.d.'s (except the e.s.d. in the dihedral angle between two l.s. planes) are estimated using the full covariance matrix. The cell e.s.d.'s are taken into account individually in the estimation of e.s.d.'s in distances, angles and torsion angles; correlations between e.s.d.'s in cell parameters are only used when they are defined by crystal symmetry. An approximate (isotropic) treatment of cell e.s.d.'s is used for estimating e.s.d.'s involving l.s. planes.
Refinement. Refinement of *F*^2^ against ALL reflections. The weighted *R*-factor *wR* and goodness of fit *S* are based on *F*^2^, conventional *R*-factors *R* are based on *F*, with *F* set to zero for negative *F*^2^. The threshold expression of *F*^2^ > σ(*F*^2^) is used only for calculating *R*-factors(gt) *etc*. and is not relevant to the choice of reflections for refinement. *R*-factors based on *F*^2^ are statistically about twice as large as those based on *F*, and *R*- factors based on ALL data will be even larger.

### Fractional atomic coordinates and isotropic or equivalent isotropic displacement parameters (Å^2^)

**Table:**

	*x*	*y*	*z*	*U*_iso_*/*U*_eq_	
S1A	0.07844 (3)	0.44292 (5)	0.10629 (3)	0.03707 (18)	
S2A	−0.03750 (3)	0.44370 (7)	0.06515 (4)	0.0536 (2)	
O1A	0.06586 (9)	0.70694 (15)	0.16755 (9)	0.0470 (5)	
O2A	0.20088 (10)	0.4378 (2)	0.07926 (13)	0.0761 (8)	
N1A	0.19449 (11)	0.6383 (2)	0.14698 (13)	0.0626 (9)	
N2A	0.17752 (12)	0.5500 (2)	0.00962 (13)	0.0627 (8)	
H2A	0.1866	0.5146	−0.0192	0.075*	
C1A	0.10975 (11)	0.5525 (2)	0.13913 (12)	0.0336 (6)	
C2A	0.15768 (11)	0.6004 (2)	0.10277 (13)	0.0417 (7)	
C3A	0.19671 (15)	0.5637 (3)	0.19186 (17)	0.0696 (11)	
H3A1	0.2116	0.5920	0.2265	0.083*	
H3A2	0.2190	0.5068	0.1809	0.083*	
C4A	0.13652 (13)	0.5336 (2)	0.19913 (13)	0.0442 (7)	
H4A	0.1202	0.5828	0.2248	0.053*	
C5A	0.06213 (11)	0.6272 (2)	0.14331 (12)	0.0348 (6)	
N6A	0.01560 (9)	0.59483 (18)	0.11580 (11)	0.0411 (6)	
H6A	−0.0125	0.6335	0.1117	0.049*	
C7A	0.01507 (11)	0.4997 (2)	0.09515 (12)	0.0345 (6)	
C8A	0.18171 (12)	0.5186 (3)	0.06360 (16)	0.0538 (9)	
C9A	0.15640 (13)	0.6479 (3)	0.00619 (16)	0.0534 (9)	
C10A	0.15109 (15)	0.7069 (3)	−0.04149 (17)	0.0685 (11)	
H10A	0.1581	0.6818	−0.0774	0.082*	
C11A	0.13493 (17)	0.8045 (4)	−0.0334 (2)	0.0822 (14)	
H11A	0.1313	0.8463	−0.0646	0.099*	
C12A	0.12411 (18)	0.8418 (3)	0.0193 (2)	0.0785 (13)	
H12A	0.1135	0.9083	0.0234	0.094*	
C13A	0.12889 (15)	0.7803 (3)	0.06675 (18)	0.0624 (10)	
H13A	0.1220	0.8057	0.1027	0.075*	
C14A	0.14398 (12)	0.6817 (2)	0.05987 (14)	0.0446 (7)	
C15A	0.24819 (17)	0.6718 (4)	0.1271 (2)	0.110 (2)	
H15A	0.2696	0.6960	0.1586	0.165*	
H15B	0.2433	0.7246	0.1000	0.165*	
H15C	0.2670	0.6168	0.1096	0.165*	
C16A	0.12790 (14)	0.4324 (2)	0.22578 (13)	0.0480 (8)	
C17A	0.0875 (2)	0.4210 (4)	0.26582 (18)	0.0935 (10)	
H17A	0.0648	0.4747	0.2748	0.112*	
C18A	0.0803 (2)	0.3289 (4)	0.29305 (19)	0.0935 (10)	
H18A	0.0530	0.3222	0.3203	0.112*	
C19A	0.1121 (2)	0.2507 (4)	0.28035 (19)	0.0935 (10)	
H19A	0.1068	0.1901	0.2988	0.112*	
C20A	0.1516 (2)	0.2587 (3)	0.2412 (2)	0.0909 (16)	
H20A	0.1734	0.2036	0.2327	0.109*	
C21A	0.16001 (17)	0.3502 (3)	0.21300 (18)	0.0696 (11)	
H21A	0.1873	0.3553	0.1857	0.084*	
S1B	0.36499 (4)	0.44011 (6)	0.16906 (4)	0.0522 (2)	
S2B	0.31112 (5)	0.44048 (10)	0.28093 (5)	0.0892 (4)	
O1B	0.43828 (9)	0.19100 (16)	0.19970 (9)	0.0503 (6)	
O2B	0.36139 (11)	0.43102 (18)	0.03885 (10)	0.0619 (7)	
N1B	0.42929 (9)	0.23907 (18)	0.06498 (10)	0.0371 (6)	
N2B	0.29280 (11)	0.3182 (2)	0.05743 (12)	0.0552 (7)	
H2B	0.2658	0.3492	0.0414	0.066*	
C1B	0.40734 (12)	0.3350 (2)	0.14583 (12)	0.0382 (7)	
C2B	0.38050 (11)	0.2770 (2)	0.09430 (12)	0.0353 (6)	
C3B	0.46916 (13)	0.3211 (2)	0.06459 (13)	0.0462 (8)	
H3B1	0.4583	0.3727	0.0379	0.055*	
H3B2	0.5057	0.2972	0.0550	0.055*	
C4B	0.46771 (12)	0.3603 (2)	0.12547 (13)	0.0419 (7)	
H4B	0.4925	0.3170	0.1475	0.050*	
C5B	0.41038 (12)	0.2663 (2)	0.19733 (12)	0.0403 (7)	
N6B	0.37778 (11)	0.2991 (2)	0.24075 (11)	0.0526 (7)	
H6B	0.3745	0.2646	0.2713	0.063*	
C7B	0.35061 (14)	0.3880 (3)	0.23427 (13)	0.0531 (9)	
C8B	0.34507 (14)	0.3535 (2)	0.05928 (13)	0.0442 (8)	
C9B	0.28757 (13)	0.2259 (3)	0.08442 (14)	0.0522 (9)	
C10B	0.24089 (16)	0.1667 (4)	0.08728 (17)	0.0769 (13)	
H10B	0.2071	0.1886	0.0732	0.092*	
C11B	0.24680 (18)	0.0736 (4)	0.11201 (19)	0.0845 (14)	
H11B	0.2163	0.0317	0.1143	0.101*	
C12B	0.29666 (18)	0.0413 (3)	0.13330 (16)	0.0683 (11)	
H12B	0.2993	−0.0218	0.1498	0.082*	
C13B	0.34306 (14)	0.1015 (3)	0.13039 (14)	0.0513 (8)	
H13B	0.3769	0.0789	0.1441	0.062*	
C14B	0.33827 (12)	0.1962 (2)	0.10669 (12)	0.0404 (7)	
C15B	0.41752 (14)	0.1977 (3)	0.00892 (13)	0.0522 (8)	
H15D	0.4512	0.1748	−0.0078	0.078*	
H15E	0.4013	0.2485	−0.0146	0.078*	
H15F	0.3924	0.1426	0.0124	0.078*	
C16B	0.48891 (14)	0.4658 (2)	0.13328 (15)	0.0492 (8)	
C17B	0.5268 (3)	0.4834 (4)	0.1750 (3)	0.1263 (14)	
H17B	0.5374	0.4318	0.1992	0.152*	
C18B	0.5496 (3)	0.5779 (4)	0.1817 (3)	0.1263 (14)	
H18B	0.5762	0.5879	0.2096	0.152*	
C19B	0.5342 (3)	0.6552 (4)	0.1489 (3)	0.1263 (14)	
H19B	0.5487	0.7187	0.1551	0.152*	
C20B	0.49755 (19)	0.6390 (3)	0.1072 (2)	0.0805 (13)	
H20B	0.4872	0.6916	0.0836	0.097*	
C21B	0.47467 (17)	0.5443 (3)	0.09865 (17)	0.0669 (10)	
H21B	0.4496	0.5342	0.0692	0.080*	

### Atomic displacement parameters (Å^2^)

**Table:**

	*U*^11^	*U*^22^	*U*^33^	*U*^12^	*U*^13^	*U*^23^
S1A	0.0367 (4)	0.0316 (4)	0.0428 (4)	0.0071 (3)	−0.0041 (3)	−0.0068 (3)
S2A	0.0362 (4)	0.0512 (5)	0.0733 (6)	−0.0032 (4)	−0.0057 (4)	−0.0160 (5)
O1A	0.0530 (13)	0.0342 (12)	0.0536 (13)	0.0066 (10)	−0.0067 (10)	−0.0132 (10)
O2A	0.0543 (15)	0.0664 (18)	0.108 (2)	0.0300 (13)	0.0262 (14)	0.0231 (16)
N1A	0.0485 (16)	0.063 (2)	0.076 (2)	−0.0201 (14)	−0.0237 (15)	0.0221 (17)
N2A	0.0622 (18)	0.064 (2)	0.063 (2)	0.0055 (16)	0.0258 (15)	0.0041 (16)
C1A	0.0325 (13)	0.0304 (15)	0.0377 (15)	0.0049 (11)	−0.0039 (11)	0.0008 (12)
C2A	0.0297 (14)	0.0445 (18)	0.0510 (19)	0.0033 (12)	−0.0011 (13)	0.0108 (15)
C3A	0.058 (2)	0.074 (3)	0.076 (3)	−0.0142 (19)	−0.0321 (19)	0.023 (2)
C4A	0.0525 (18)	0.0396 (18)	0.0405 (17)	−0.0004 (14)	−0.0125 (14)	0.0018 (14)
C5A	0.0382 (15)	0.0332 (16)	0.0330 (15)	0.0017 (12)	0.0022 (12)	−0.0032 (12)
N6A	0.0308 (12)	0.0352 (14)	0.0573 (17)	0.0080 (10)	−0.0025 (11)	−0.0109 (12)
C7A	0.0341 (14)	0.0316 (16)	0.0379 (16)	−0.0008 (11)	0.0053 (12)	−0.0044 (12)
C8A	0.0316 (16)	0.055 (2)	0.075 (3)	0.0080 (15)	0.0158 (16)	0.0141 (19)
C9A	0.0428 (18)	0.057 (2)	0.061 (2)	−0.0046 (16)	0.0045 (16)	0.0148 (18)
C10A	0.061 (2)	0.089 (3)	0.056 (2)	−0.010 (2)	0.0069 (18)	0.022 (2)
C11A	0.069 (3)	0.089 (3)	0.089 (3)	0.001 (2)	0.008 (2)	0.049 (3)
C12A	0.079 (3)	0.055 (3)	0.101 (4)	0.004 (2)	0.009 (3)	0.038 (3)
C13A	0.063 (2)	0.049 (2)	0.076 (3)	−0.0011 (17)	0.0058 (19)	0.0151 (19)
C14A	0.0369 (16)	0.0448 (19)	0.052 (2)	−0.0002 (13)	−0.0001 (14)	0.0135 (15)
C15A	0.055 (2)	0.138 (5)	0.137 (4)	−0.049 (3)	−0.032 (3)	0.063 (4)
C16A	0.063 (2)	0.0434 (19)	0.0368 (17)	−0.0033 (16)	−0.0166 (15)	0.0032 (14)
C17A	0.146 (3)	0.0746 (19)	0.0599 (16)	−0.0371 (19)	0.0032 (17)	0.0009 (14)
C18A	0.146 (3)	0.0746 (19)	0.0599 (16)	−0.0371 (19)	0.0032 (17)	0.0009 (14)
C19A	0.146 (3)	0.0746 (19)	0.0599 (16)	−0.0371 (19)	0.0032 (17)	0.0009 (14)
C20A	0.114 (4)	0.046 (2)	0.112 (4)	0.005 (2)	−0.057 (3)	0.007 (2)
C21A	0.073 (3)	0.050 (2)	0.085 (3)	0.0097 (19)	−0.014 (2)	0.014 (2)
S1B	0.0704 (6)	0.0407 (5)	0.0455 (5)	0.0290 (4)	0.0036 (4)	−0.0023 (4)
S2B	0.1098 (9)	0.1043 (9)	0.0537 (6)	0.0609 (8)	0.0190 (6)	−0.0113 (6)
O1B	0.0636 (14)	0.0409 (13)	0.0464 (13)	0.0242 (11)	0.0049 (11)	0.0072 (10)
O2B	0.0807 (17)	0.0452 (14)	0.0596 (16)	0.0130 (12)	−0.0078 (13)	0.0167 (12)
N1B	0.0390 (13)	0.0367 (14)	0.0357 (13)	0.0064 (10)	0.0065 (10)	−0.0058 (11)
N2B	0.0428 (15)	0.070 (2)	0.0530 (17)	0.0194 (14)	−0.0034 (13)	0.0125 (15)
C1B	0.0466 (16)	0.0313 (15)	0.0367 (16)	0.0186 (13)	0.0028 (13)	0.0009 (12)
C2B	0.0375 (15)	0.0351 (16)	0.0335 (15)	0.0157 (12)	0.0023 (12)	0.0021 (12)
C3B	0.0466 (17)	0.0453 (19)	0.0470 (19)	0.0048 (14)	0.0104 (14)	−0.0013 (15)
C4B	0.0417 (16)	0.0375 (17)	0.0465 (18)	0.0082 (13)	−0.0034 (13)	0.0006 (14)
C5B	0.0494 (17)	0.0396 (17)	0.0320 (16)	0.0141 (14)	−0.0033 (13)	−0.0023 (13)
N6B	0.0723 (19)	0.0540 (17)	0.0315 (14)	0.0285 (15)	0.0073 (13)	0.0031 (12)
C7B	0.066 (2)	0.055 (2)	0.0382 (18)	0.0278 (17)	−0.0050 (16)	−0.0074 (15)
C8B	0.0545 (19)	0.0451 (19)	0.0329 (17)	0.0184 (15)	−0.0013 (14)	−0.0007 (14)
C9B	0.0422 (18)	0.072 (2)	0.0422 (19)	0.0081 (17)	0.0079 (14)	0.0062 (17)
C10B	0.045 (2)	0.123 (4)	0.063 (3)	−0.007 (2)	0.0050 (18)	0.021 (3)
C11B	0.065 (3)	0.118 (4)	0.071 (3)	−0.034 (3)	0.012 (2)	0.015 (3)
C12B	0.081 (3)	0.067 (3)	0.058 (2)	−0.019 (2)	0.014 (2)	0.011 (2)
C13B	0.057 (2)	0.049 (2)	0.049 (2)	0.0055 (16)	0.0081 (16)	0.0036 (16)
C14B	0.0410 (16)	0.0455 (18)	0.0348 (16)	0.0074 (14)	0.0074 (13)	0.0004 (14)
C15B	0.061 (2)	0.054 (2)	0.0409 (19)	0.0061 (17)	0.0089 (15)	−0.0134 (16)
C16B	0.057 (2)	0.0360 (18)	0.055 (2)	0.0039 (14)	−0.0059 (16)	−0.0008 (15)
C17B	0.164 (3)	0.0598 (18)	0.154 (3)	−0.022 (2)	−0.091 (3)	0.0004 (19)
C18B	0.164 (3)	0.0598 (18)	0.154 (3)	−0.022 (2)	−0.091 (3)	0.0004 (19)
C19B	0.164 (3)	0.0598 (18)	0.154 (3)	−0.022 (2)	−0.091 (3)	0.0004 (19)
C20B	0.103 (3)	0.043 (2)	0.096 (3)	0.002 (2)	0.011 (3)	0.013 (2)
C21B	0.085 (3)	0.043 (2)	0.073 (3)	−0.0003 (19)	−0.013 (2)	0.0076 (19)

### Geometric parameters (Å, °)

**Table:**

S1A—C7A	1.733 (3)	S1B—C7B	1.728 (4)
S1A—C1A	1.820 (3)	S1B—C1B	1.826 (3)
S2A—C7A	1.636 (3)	S2B—C7B	1.625 (3)
O1A—C5A	1.213 (3)	O1B—C5B	1.214 (3)
O2A—C8A	1.232 (4)	O2B—C8B	1.210 (4)
N1A—C3A	1.456 (4)	N1B—C15B	1.462 (4)
N1A—C15A	1.458 (5)	N1B—C3B	1.462 (4)
N1A—C2A	1.460 (4)	N1B—C2B	1.466 (3)
N2A—C8A	1.346 (5)	N2B—C8B	1.353 (4)
N2A—C9A	1.406 (4)	N2B—C9B	1.394 (4)
N2A—H2A	0.8600	N2B—H2B	0.8600
C1A—C5A	1.530 (4)	C1B—C5B	1.525 (4)
C1A—C4A	1.575 (4)	C1B—C2B	1.580 (4)
C1A—C2A	1.586 (4)	C1B—C4B	1.581 (4)
C2A—C14A	1.520 (4)	C2B—C14B	1.518 (4)
C2A—C8A	1.549 (5)	C2B—C8B	1.568 (4)
C3A—C4A	1.526 (5)	C3B—C4B	1.531 (4)
C3A—H3A1	0.9700	C3B—H3B1	0.9700
C3A—H3A2	0.9700	C3B—H3B2	0.9700
C4A—C16A	1.506 (4)	C4B—C16B	1.511 (4)
C4A—H4A	0.9800	C4B—H4B	0.9800
C5A—N6A	1.368 (4)	C5B—N6B	1.372 (4)
N6A—C7A	1.361 (4)	N6B—C7B	1.367 (4)
N6A—H6A	0.8600	N6B—H6B	0.8600
C9A—C10A	1.380 (5)	C9B—C10B	1.383 (5)
C9A—C14A	1.382 (5)	C9B—C14B	1.392 (4)
C10A—C11A	1.376 (6)	C10B—C11B	1.381 (6)
C10A—H10A	0.9300	C10B—H10B	0.9300
C11A—C12A	1.367 (6)	C11B—C12B	1.376 (6)
C11A—H11A	0.9300	C11B—H11B	0.9300
C12A—C13A	1.394 (5)	C12B—C13B	1.386 (5)
C12A—H12A	0.9300	C12B—H12B	0.9300
C13A—C14A	1.378 (5)	C13B—C14B	1.387 (4)
C13A—H13A	0.9300	C13B—H13B	0.9300
C15A—H15A	0.9600	C15B—H15D	0.9600
C15A—H15B	0.9600	C15B—H15E	0.9600
C15A—H15C	0.9600	C15B—H15F	0.9600
C16A—C17A	1.376 (6)	C16B—C17B	1.363 (6)
C16A—C21A	1.381 (5)	C16B—C21B	1.372 (5)
C17A—C18A	1.399 (6)	C17B—C18B	1.386 (6)
C17A—H17A	0.9300	C17B—H17B	0.9300
C18A—C19A	1.334 (7)	C18B—C19B	1.342 (7)
C18A—H18A	0.9300	C18B—H18B	0.9300
C19A—C20A	1.341 (7)	C19B—C20B	1.341 (6)
C19A—H19A	0.9300	C19B—H19B	0.9300
C20A—C21A	1.408 (6)	C20B—C21B	1.396 (5)
C20A—H20A	0.9300	C20B—H20B	0.9300
C21A—H21A	0.9300	C21B—H21B	0.9300
			
C7A—S1A—C1A	94.56 (12)	C7B—S1B—C1B	94.37 (14)
C3A—N1A—C15A	114.6 (3)	C15B—N1B—C3B	113.7 (2)
C3A—N1A—C2A	107.6 (3)	C15B—N1B—C2B	113.9 (2)
C15A—N1A—C2A	114.8 (3)	C3B—N1B—C2B	106.3 (2)
C8A—N2A—C9A	111.7 (3)	C8B—N2B—C9B	112.4 (3)
C8A—N2A—H2A	124.2	C8B—N2B—H2B	123.8
C9A—N2A—H2A	124.2	C9B—N2B—H2B	123.8
C5A—C1A—C4A	110.7 (2)	C5B—C1B—C2B	109.7 (2)
C5A—C1A—C2A	109.2 (2)	C5B—C1B—C4B	109.3 (2)
C4A—C1A—C2A	104.7 (2)	C2B—C1B—C4B	104.3 (2)
C5A—C1A—S1A	103.84 (18)	C5B—C1B—S1B	104.28 (19)
C4A—C1A—S1A	115.01 (19)	C2B—C1B—S1B	112.30 (18)
C2A—C1A—S1A	113.5 (2)	C4B—C1B—S1B	116.9 (2)
N1A—C2A—C14A	111.0 (3)	N1B—C2B—C14B	113.2 (2)
N1A—C2A—C8A	116.2 (3)	N1B—C2B—C8B	114.7 (2)
C14A—C2A—C8A	100.8 (3)	C14B—C2B—C8B	101.4 (2)
N1A—C2A—C1A	101.5 (2)	N1B—C2B—C1B	101.8 (2)
C14A—C2A—C1A	119.5 (2)	C14B—C2B—C1B	118.4 (2)
C8A—C2A—C1A	108.6 (2)	C8B—C2B—C1B	107.9 (2)
N1A—C3A—C4A	103.4 (3)	N1B—C3B—C4B	103.3 (2)
N1A—C3A—H3A1	111.1	N1B—C3B—H3B1	111.1
C4A—C3A—H3A1	111.1	C4B—C3B—H3B1	111.1
N1A—C3A—H3A2	111.1	N1B—C3B—H3B2	111.1
C4A—C3A—H3A2	111.1	C4B—C3B—H3B2	111.1
H3A1—C3A—H3A2	109.0	H3B1—C3B—H3B2	109.1
C16A—C4A—C3A	114.8 (3)	C16B—C4B—C3B	115.1 (3)
C16A—C4A—C1A	117.5 (2)	C16B—C4B—C1B	118.5 (2)
C3A—C4A—C1A	104.2 (3)	C3B—C4B—C1B	103.9 (2)
C16A—C4A—H4A	106.5	C16B—C4B—H4B	106.2
C3A—C4A—H4A	106.5	C3B—C4B—H4B	106.2
C1A—C4A—H4A	106.5	C1B—C4B—H4B	106.2
O1A—C5A—N6A	124.1 (3)	O1B—C5B—N6B	123.7 (3)
O1A—C5A—C1A	123.4 (3)	O1B—C5B—C1B	124.0 (3)
N6A—C5A—C1A	112.6 (2)	N6B—C5B—C1B	112.3 (2)
C7A—N6A—C5A	118.1 (2)	C7B—N6B—C5B	118.3 (3)
C7A—N6A—H6A	121.0	C7B—N6B—H6B	120.9
C5A—N6A—H6A	121.0	C5B—N6B—H6B	120.9
N6A—C7A—S2A	126.0 (2)	N6B—C7B—S2B	125.8 (3)
N6A—C7A—S1A	110.4 (2)	N6B—C7B—S1B	110.5 (2)
S2A—C7A—S1A	123.60 (17)	S2B—C7B—S1B	123.7 (2)
O2A—C8A—N2A	125.7 (4)	O2B—C8B—N2B	126.5 (3)
O2A—C8A—C2A	125.6 (3)	O2B—C8B—C2B	126.1 (3)
N2A—C8A—C2A	108.7 (3)	N2B—C8B—C2B	107.4 (3)
C10A—C9A—C14A	122.9 (4)	C10B—C9B—C14B	122.7 (4)
C10A—C9A—N2A	127.6 (4)	C10B—C9B—N2B	127.2 (3)
C14A—C9A—N2A	109.4 (3)	C14B—C9B—N2B	110.0 (3)
C11A—C10A—C9A	116.9 (4)	C11B—C10B—C9B	116.9 (4)
C11A—C10A—H10A	121.6	C11B—C10B—H10B	121.5
C9A—C10A—H10A	121.6	C9B—C10B—H10B	121.5
C12A—C11A—C10A	122.0 (4)	C12B—C11B—C10B	121.7 (4)
C12A—C11A—H11A	119.0	C12B—C11B—H11B	119.1
C10A—C11A—H11A	119.0	C10B—C11B—H11B	119.1
C11A—C12A—C13A	120.1 (4)	C11B—C12B—C13B	120.7 (4)
C11A—C12A—H12A	120.0	C11B—C12B—H12B	119.6
C13A—C12A—H12A	120.0	C13B—C12B—H12B	119.6
C14A—C13A—C12A	119.3 (4)	C12B—C13B—C14B	118.9 (3)
C14A—C13A—H13A	120.4	C12B—C13B—H13B	120.5
C12A—C13A—H13A	120.4	C14B—C13B—H13B	120.5
C13A—C14A—C9A	118.7 (3)	C13B—C14B—C9B	118.9 (3)
C13A—C14A—C2A	131.4 (3)	C13B—C14B—C2B	132.2 (3)
C9A—C14A—C2A	109.3 (3)	C9B—C14B—C2B	108.6 (3)
N1A—C15A—H15A	109.5	N1B—C15B—H15D	109.5
N1A—C15A—H15B	109.5	N1B—C15B—H15E	109.5
H15A—C15A—H15B	109.5	H15D—C15B—H15E	109.5
N1A—C15A—H15C	109.5	N1B—C15B—H15F	109.5
H15A—C15A—H15C	109.5	H15D—C15B—H15F	109.5
H15B—C15A—H15C	109.5	H15E—C15B—H15F	109.5
C17A—C16A—C21A	117.9 (4)	C17B—C16B—C21B	117.7 (4)
C17A—C16A—C4A	119.3 (3)	C17B—C16B—C4B	118.4 (3)
C21A—C16A—C4A	122.8 (3)	C21B—C16B—C4B	123.8 (3)
C16A—C17A—C18A	120.4 (5)	C16B—C17B—C18B	120.4 (5)
C16A—C17A—H17A	119.8	C16B—C17B—H17B	119.8
C18A—C17A—H17A	119.8	C18B—C17B—H17B	119.8
C19A—C18A—C17A	120.7 (5)	C19B—C18B—C17B	121.8 (5)
C19A—C18A—H18A	119.6	C19B—C18B—H18B	119.1
C17A—C18A—H18A	119.6	C17B—C18B—H18B	119.1
C18A—C19A—C20A	120.7 (5)	C20B—C19B—C18B	118.7 (5)
C18A—C19A—H19A	119.7	C20B—C19B—H19B	120.7
C20A—C19A—H19A	119.7	C18B—C19B—H19B	120.7
C19A—C20A—C21A	120.2 (5)	C19B—C20B—C21B	120.8 (4)
C19A—C20A—H20A	119.9	C19B—C20B—H20B	119.6
C21A—C20A—H20A	119.9	C21B—C20B—H20B	119.6
C16A—C21A—C20A	120.2 (4)	C16B—C21B—C20B	120.6 (4)
C16A—C21A—H21A	119.9	C16B—C21B—H21B	119.7
C20A—C21A—H21A	119.9	C20B—C21B—H21B	119.7
			
C7A—S1A—C1A—C5A	5.1 (2)	C7B—S1B—C1B—C5B	−4.6 (2)
C7A—S1A—C1A—C4A	126.1 (2)	C7B—S1B—C1B—C2B	114.0 (2)
C7A—S1A—C1A—C2A	−113.4 (2)	C7B—S1B—C1B—C4B	−125.4 (2)
C3A—N1A—C2A—C14A	168.7 (3)	C15B—N1B—C2B—C14B	63.0 (3)
C15A—N1A—C2A—C14A	−62.4 (4)	C3B—N1B—C2B—C14B	−170.9 (2)
C3A—N1A—C2A—C8A	−76.9 (4)	C15B—N1B—C2B—C8B	−52.7 (3)
C15A—N1A—C2A—C8A	51.9 (4)	C3B—N1B—C2B—C8B	73.4 (3)
C3A—N1A—C2A—C1A	40.7 (3)	C15B—N1B—C2B—C1B	−168.9 (2)
C15A—N1A—C2A—C1A	169.6 (3)	C3B—N1B—C2B—C1B	−42.8 (3)
C5A—C1A—C2A—N1A	97.5 (3)	C5B—C1B—C2B—N1B	−94.1 (3)
C4A—C1A—C2A—N1A	−21.0 (3)	C4B—C1B—C2B—N1B	23.0 (3)
S1A—C1A—C2A—N1A	−147.2 (2)	S1B—C1B—C2B—N1B	150.45 (19)
C5A—C1A—C2A—C14A	−24.9 (4)	C5B—C1B—C2B—C14B	30.6 (3)
C4A—C1A—C2A—C14A	−143.4 (3)	C4B—C1B—C2B—C14B	147.7 (2)
S1A—C1A—C2A—C14A	90.4 (3)	S1B—C1B—C2B—C14B	−84.8 (3)
C5A—C1A—C2A—C8A	−139.6 (2)	C5B—C1B—C2B—C8B	144.9 (2)
C4A—C1A—C2A—C8A	101.9 (3)	C4B—C1B—C2B—C8B	−98.1 (2)
S1A—C1A—C2A—C8A	−24.3 (3)	S1B—C1B—C2B—C8B	29.4 (3)
C15A—N1A—C3A—C4A	−173.4 (4)	C15B—N1B—C3B—C4B	172.0 (2)
C2A—N1A—C3A—C4A	−44.4 (4)	C2B—N1B—C3B—C4B	45.8 (3)
N1A—C3A—C4A—C16A	157.7 (3)	N1B—C3B—C4B—C16B	−159.4 (2)
N1A—C3A—C4A—C1A	27.7 (4)	N1B—C3B—C4B—C1B	−28.3 (3)
C5A—C1A—C4A—C16A	110.3 (3)	C5B—C1B—C4B—C16B	−110.6 (3)
C2A—C1A—C4A—C16A	−132.2 (3)	C2B—C1B—C4B—C16B	132.2 (3)
S1A—C1A—C4A—C16A	−7.0 (4)	S1B—C1B—C4B—C16B	7.5 (4)
C5A—C1A—C4A—C3A	−121.4 (3)	C5B—C1B—C4B—C3B	120.3 (3)
C2A—C1A—C4A—C3A	−3.9 (3)	C2B—C1B—C4B—C3B	3.1 (3)
S1A—C1A—C4A—C3A	121.3 (3)	S1B—C1B—C4B—C3B	−121.6 (2)
C4A—C1A—C5A—O1A	49.6 (4)	C2B—C1B—C5B—O1B	64.7 (4)
C2A—C1A—C5A—O1A	−65.1 (4)	C4B—C1B—C5B—O1B	−49.1 (4)
S1A—C1A—C5A—O1A	173.5 (2)	S1B—C1B—C5B—O1B	−174.8 (3)
C4A—C1A—C5A—N6A	−131.7 (3)	C2B—C1B—C5B—N6B	−115.0 (3)
C2A—C1A—C5A—N6A	113.6 (3)	C4B—C1B—C5B—N6B	131.1 (3)
S1A—C1A—C5A—N6A	−7.8 (3)	S1B—C1B—C5B—N6B	5.4 (3)
O1A—C5A—N6A—C7A	−173.6 (3)	O1B—C5B—N6B—C7B	176.4 (3)
C1A—C5A—N6A—C7A	7.7 (4)	C1B—C5B—N6B—C7B	−3.8 (4)
C5A—N6A—C7A—S2A	175.3 (2)	C5B—N6B—C7B—S2B	−178.8 (3)
C5A—N6A—C7A—S1A	−3.4 (3)	C5B—N6B—C7B—S1B	0.0 (4)
C1A—S1A—C7A—N6A	−1.5 (2)	C1B—S1B—C7B—N6B	3.0 (3)
C1A—S1A—C7A—S2A	179.75 (19)	C1B—S1B—C7B—S2B	−178.2 (3)
C9A—N2A—C8A—O2A	−177.0 (3)	C9B—N2B—C8B—O2B	179.2 (3)
C9A—N2A—C8A—C2A	3.8 (4)	C9B—N2B—C8B—C2B	−2.3 (4)
N1A—C2A—C8A—O2A	57.2 (4)	N1B—C2B—C8B—O2B	−56.4 (4)
C14A—C2A—C8A—O2A	177.2 (3)	C14B—C2B—C8B—O2B	−178.7 (3)
C1A—C2A—C8A—O2A	−56.4 (4)	C1B—C2B—C8B—O2B	56.2 (4)
N1A—C2A—C8A—N2A	−123.6 (3)	N1B—C2B—C8B—N2B	125.1 (3)
C14A—C2A—C8A—N2A	−3.5 (3)	C14B—C2B—C8B—N2B	2.8 (3)
C1A—C2A—C8A—N2A	122.8 (3)	C1B—C2B—C8B—N2B	−122.3 (3)
C8A—N2A—C9A—C10A	173.9 (3)	C8B—N2B—C9B—C10B	−175.8 (4)
C8A—N2A—C9A—C14A	−2.4 (4)	C8B—N2B—C9B—C14B	0.8 (4)
C14A—C9A—C10A—C11A	2.9 (5)	C14B—C9B—C10B—C11B	−2.0 (6)
N2A—C9A—C10A—C11A	−172.9 (4)	N2B—C9B—C10B—C11B	174.2 (4)
C9A—C10A—C11A—C12A	−0.5 (6)	C9B—C10B—C11B—C12B	0.5 (7)
C10A—C11A—C12A—C13A	−0.5 (7)	C10B—C11B—C12B—C13B	−0.3 (7)
C11A—C12A—C13A—C14A	−0.7 (6)	C11B—C12B—C13B—C14B	1.3 (6)
C12A—C13A—C14A—C9A	3.0 (5)	C12B—C13B—C14B—C9B	−2.7 (5)
C12A—C13A—C14A—C2A	173.4 (3)	C12B—C13B—C14B—C2B	−176.1 (3)
C10A—C9A—C14A—C13A	−4.1 (5)	C10B—C9B—C14B—C13B	3.1 (5)
N2A—C9A—C14A—C13A	172.3 (3)	N2B—C9B—C14B—C13B	−173.7 (3)
C10A—C9A—C14A—C2A	−176.6 (3)	C10B—C9B—C14B—C2B	178.0 (3)
N2A—C9A—C14A—C2A	−0.1 (4)	N2B—C9B—C14B—C2B	1.2 (4)
N1A—C2A—C14A—C13A	−45.3 (4)	N1B—C2B—C14B—C13B	48.2 (4)
C8A—C2A—C14A—C13A	−169.0 (3)	C8B—C2B—C14B—C13B	171.5 (3)
C1A—C2A—C14A—C13A	72.2 (4)	C1B—C2B—C14B—C13B	−70.7 (4)
N1A—C2A—C14A—C9A	125.8 (3)	N1B—C2B—C14B—C9B	−125.7 (3)
C8A—C2A—C14A—C9A	2.1 (3)	C8B—C2B—C14B—C9B	−2.4 (3)
C1A—C2A—C14A—C9A	−116.6 (3)	C1B—C2B—C14B—C9B	115.4 (3)
C3A—C4A—C16A—C17A	138.1 (4)	C3B—C4B—C16B—C17B	−130.5 (5)
C1A—C4A—C16A—C17A	−98.9 (4)	C1B—C4B—C16B—C17B	105.8 (5)
C3A—C4A—C16A—C21A	−40.0 (5)	C3B—C4B—C16B—C21B	45.6 (5)
C1A—C4A—C16A—C21A	83.0 (4)	C1B—C4B—C16B—C21B	−78.1 (4)
C21A—C16A—C17A—C18A	1.1 (6)	C21B—C16B—C17B—C18B	0.1 (9)
C4A—C16A—C17A—C18A	−177.1 (4)	C4B—C16B—C17B—C18B	176.4 (5)
C16A—C17A—C18A—C19A	−0.6 (7)	C16B—C17B—C18B—C19B	2.1 (11)
C17A—C18A—C19A—C20A	0.0 (8)	C17B—C18B—C19B—C20B	−2.9 (11)
C18A—C19A—C20A—C21A	0.1 (7)	C18B—C19B—C20B—C21B	1.5 (10)
C17A—C16A—C21A—C20A	−1.0 (6)	C17B—C16B—C21B—C20B	−1.4 (7)
C4A—C16A—C21A—C20A	177.1 (3)	C4B—C16B—C21B—C20B	−177.5 (4)
C19A—C20A—C21A—C16A	0.4 (6)	C19B—C20B—C21B—C16B	0.7 (7)

### Hydrogen-bond geometry (Å, °)

**Table:**

Cg1 is the centroid of the C9*B*–C14*B* ring.

**Table:**

*D*—H···*A*	*D*—H	H···*A*	*D*···*A*	*D*—H···*A*
N2B—H2B···O2A	0.86	2.17	2.794 (3)	129
N6A—H6A···N1B^i^	0.86	2.28	3.083 (3)	156
C4B—H4B···O1A^ii^	0.98	2.36	3.290 (3)	159
N6B—H6B···O1A^iii^	0.86	2.18	2.836 (3)	133
N2A—H2A···Cg1^iv^	0.86	2.95	3.806 (3)	170

Symmetry codes: (i) *x*−1/2, *y*+1/2, *z*; (ii) *x*+1/2, *y*−1/2, *z*; (iii) −*x*+1/2, *y*−1/2, −*z*+1/2; (iv) −*x*+1/2, −*y*+1/2, −*z*.
